# Intravitreal Dexamethasone Implants for Refractory Macular Edema in Eyes with Noninfectious Uveitis

**DOI:** 10.3390/jcm10173762

**Published:** 2021-08-24

**Authors:** Eugene Yu-Chuan Kang, Sunir J. Garg, Hsi-Fu Chen, Wei-Chi Wu, Linda Yi-Hsing Chen, Hung-Da Chou, Laura Liu, Kuan-Jen Chen, Yih-Shiou Hwang

**Affiliations:** 1 Department of Ophthalmology, Chang Gung Memorial Hospital, Linkou Medical Center, Taoyuan 333, Taiwan; yckang0321@gmail.com (E.Y.-C.K.); weichi666@gmail.com (W.-C.W.); yihsing@gmail.com (L.Y.-H.C.); hdmorph@gmail.com (H.-D.C.); laurajl@gmail.com (L.L.); cgr999@gmail.com (K.-J.C.); 2 College of Medicine, Chang Gung University, Taoyuan 333, Taiwan; 3 The Retina Service of Wills Eye Hospital, Thomas Jefferson University, Philadelphia, PA 19107, USA; sunirgarg@gmail.com; 4 Department of Ophthalmology, Hsinchu MacKay Memorial Hospital, Hsinchu 300, Taiwan; aurora6th@gmail.com; 5 Department of Ophthalmology, Chang Gung Memorial Hospital, Xiamen 361000, China

**Keywords:** intravitreal dexamethasone implants, refractory macular edema, visual acuity, central retinal thickness, intraocular pressure

## Abstract

Macular edema (ME) is a common cause of visual loss among eyes with uveitis, and its management can be challenging. Steroids are an effective treatment for ME, and intravitreal dexamethasone (DEX) implants provide sustained steroid release. The purpose of this study is to evaluate intravitreal DEX implant on refractory ME in eyes with noninfectious uveitis. A retrospective study including 52 eyes of 37 patients with refractory uveitic ME was conducted from January 2011 through August 2017 at Linkou Chang Gung Memorial Hospital in Taiwan. Patients’ demographic characteristics were collected. In addition, clinical information, including corrected visual acuity (VA), intraocular pressure (IOP), and central retinal thickness (CRT) on optical coherence tomography, was recorded and analyzed. During the study period, affected eyes received a total of 110 intravitreal DEX implants (range, one to six in each eye). After the first DEX implant injection in all eyes, VA significantly improved at one and two months. CRT significantly decreased one month after a single DEX implant, and the effect lasted for six months and waned over time. Patients receiving multiple DEX implants still showed significant decreases in CRT one month after the first implant. Increases in IOP were noted one month after the DEX implant, but the IOP could be medically controlled. Intravitreal DEX implants can effectively treat refractory uveitic ME, improving both VA and CRT with an acceptable safety profile. Further studies are necessary to evaluate the effect of multiple implants and long-term outcomes.

## 1. Introduction

Noninfectious uveitis is an inflammatory ocular disease, which is the leading cause of visual impairment in developed countries and responsible for about 20% of legal blindness [1]. In addition, macular edema (ME) is a significant risk factor for visual loss in eyes with uveitis, decreasing visual acuity (VA) to <20/40 in about one-third of posterior uveitis patients [2,3].

Treatment of noninfectious uveitis is mainly focused on suppressing inflammation. Immunosuppression with medications including antimetabolites and immunomodulatory agents [4] can also control intraocular inflammation but can have systemic side effects and variable success in treating ME. Systemic, topical, periocular, and intraocular steroids have been widely used to treat uveitis due to their fast and effective anti-inflammatory properties [5,6]. Although systemic corticosteroids have been used in patients with severe uveitis, they are generally associated with side effects, including osteoporosis, hyperglycemia, arterial hypertension, weight gain, glaucoma, and cataract formation. Treatment with topical steroids has several disadvantages compared to other ways of administration, including the requirement of frequent application, preservatives-induced toxicity to the ocular surface, and the inability to access the posterior structures of the eye [7]. Both sub-tenon and intraocular steroid injections help treat uveitis and uveitis-associated ME [8]. 

Intravitreal dexamethasone (DEX) implants (Ozurdex, Allergan, Irvine, CA, USA) have been used since 2009 for the treatment of different ocular diseases such as retinal vein occlusion [9], uveitis [10], and diabetic ME [11]. The implant is a biodegradable polymer composed of a combination of 0.7 mg dexamethasone and poly (lactic-co-glycolic acid) [12]. Administered through an intravitreal injection, the implant slowly dissolves in the vitreous cavity and provides sustained release of DEX for up to six months [13,14,15]. 

A clinical trial evaluating the safety and effectiveness of DEX implants in patients with noninfectious posterior uveitis showed that, after six months, VA was significantly improved [16]. In addition, other clinical trials have shown that DEX implants are also effective in uveitic ME and for the treatment of uveitis combined with systemic therapy [13,14,15]. However, there is limited research looking into intravitreal DEX implants for refractory ME in uveitis. Therefore, the purpose of this study was to evaluate the effects of DEX intravitreal implants in the eyes of patients with refractory ME due to noninfectious uveitis.

## 2. Materials and Methods

### 2.1. Study Design and Population

A retrospective case series study was conducted at Linkou Chang Gung Memorial Hospital Uveitis Service from January 2011 to August 2017. The study included patients with DEX implantations for uveitic ME and excluded patients with active uveitis with DEX implantation. The patients without an anti-inflammatory agent before DEX implantation were also excluded. The study included a total of 110 intravitreal DEX implants administered to 52 eyes from 37 patients with ME refractory to topical and/or systemic medical treatments in inflammatory controlled noninfectious uveitis. All patients had received topical (including eye drops or posterior sub-tenon injection) or systemic steroid therapy and the refractory uveitic ME in this study was defined as the persistent ME after uveitis was completely controlled. The treatments before DEX implants are shown in Table 1; 49, 19, and 41 of the 52 eyes had systemic steroid therapy, topical steroid eye drops, and posterior sub-tenon triamcinolone injection, respectively. If ME persisted after systemic or topical steroid cessation, DEX implants were used. Of note, 34 of the 52 eyes included in the study were undergoing disease-modifying antirheumatic drug (DMARD) administration along with DEX implants. Twenty-seven patients were women, and the mean age of all participants was 37 (range, 11–73 years old) at the time of the first injection (baseline). The study was approved by the Chang Gung Memorial Hospital Institutional Review Board (No. 201302539B0) and performed in compliance with regulatory obligations, the Declaration of Helsinki, and the institutional review board.

### 2.2. Patient Information and Outcome Measurements

Patients’ characteristics and clinical information including, sex, age, uveitis-causing disease, treatment, and the number of intravitreal DEX implants, were recorded. In patients with more than one DEX implant, the interval between injections was at least six months. The outcomes under consideration in this study were best-corrected VA (BCVA), central retinal thickness (CRT), and intraocular pressure (IOP). BCVA measurements were converted to the LogMAR (logarithm of the minimum angle of resolution) VA scale for statistical analysis. CRT was measured by optical coherence tomography (OCT) (Spectralis, Heidelberg Engineering, Franklin, MA, USA) and determined as the average retinal thickness within the central fovea circle of a 500 μm radius. IOP was measured by tonometry (Tono-Pen, Reichert, Depew, NY, USA). The outcomes were measured at baseline and one, two, three, and six months after the implant. The baseline means for LogMAR VA, CRT, and IOP were 0.81 (range, 0.3–2.0), 507.5 µm (range, 267–792 µm), and 13.3 mmHg (range, 6–25 mmHg), respectively.

### 2.3. Statistical Analyses

For the time-varying changes in ocular biomarkers (i.e., VA, CRT, and IOP) that were repeatedly measured during the follow-up period, a mixed model analysis was performed. The analysis considered the different groups, the assessment time points, and the interaction between these two parameters as fixed effects, and individual patients as random effects [17]. In the subgroup analysis of different implantation number, the paired-sample t-test was used to evaluate changes in LogMAR VA, IOP, and CRT values between baseline and other time points in cases of normal distribution. No missing data substitutions were made; missing data were excluded pairwise in all tests. A *p*-value < 0.05 was regarded as statistically significant. Statistical analyses were calculated using SPSS Statistics for Windows software version 24 (IBM Corporation, Armonk, NY, USA).

## 3. Results

### 3.1. Demographic and Population Characteristics

Demographic and baseline characteristics of the study population are shown in Table 1. In this study, 73.0% of the patients were women, and the most common inflammatory diseases were idiopathic uveitis (34.6%), followed by Behcet’s disease (17.3%), juvenile idiopathic arthritis (13.5%), Vogt–Koyanagi–Harada syndrome (9.6%), and acute anterior uveitis (9.6%) (Table 1). In this study, 94.2% of the patients received systemic steroid therapy, 36.5% received topical steroid eye drops prior to dexamethasone implantation, 78.8% of the patients received previous posterior sub-tenon triamcinolone (Triamcinolone Suspended Injection, Tai Yu Chemical & Pharmaceutical Co., Hsinchu, Taiwan) injection, 65.4% received previous disease-modifying antirheumatic drugs, and 64.7% were pseudophakic at baseline. During the mean follow-up period of 11.54 months, 24 eyes (46.2%) received one implant, 15 eyes (28.8%) received two implants, seven eyes (13.5%) received three implants, and three eyes (5.8%) received four or more implants. The criteria for multiple implants included (1) macular edema was not related to other pathology such as epiretinal membrane formation, (2) previous injections had therapeutic responses to macular edema without severe adverse effects such as retinal detachment, (3) the patient agreed and could afford to have the dexamethasone implant. The reinjection of dexamethasone implant was applied at recurrence of macular edema, 6 months after the previous injection. Baseline values of the outcome measurements are also reported in Table 1.

### 3.2. A Single DEX Implant Improved VA and Decreased CRT

**Figure 1:**
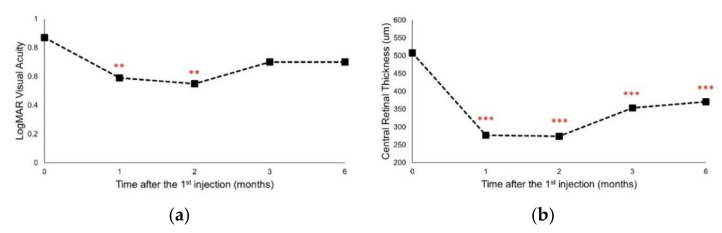
The change in (**a**) LogMAR visual acuity and (**b**) central retinal thickness from baseline after the 1st intravitreal dexamethasone implantation at different time points. Values represent mean ± SD. *p* < 0.05 were considered statistically different. ** *p* < 0.01, *** *p* < 0.001.

Compared to baseline, significant VA improvement, expressed as LogMAR VA, occurred at both one and two months after the first DEX implant (*p* < 0.01 vs. baseline; Figure 1a). There was also a significant decrease in CRT one month after the first DEX implant (*p* < 0.001), and it remained significantly lower for six months when compared to baseline values (all *p* < 0.001; Figure 1b).

### 3.3. Multiple DEX Implants Decrease CRT but Have No Change on VA

**Figure 2:**
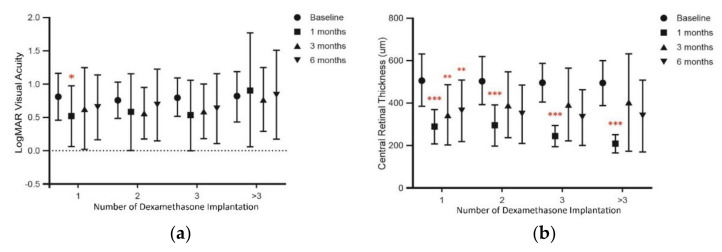
The change in (**a**) LogMAR visual acuity and (**b**) central retinal thickness from baseline after the intravitreal dexamethasone implantation based on the number of implantations at different time points. Values represent mean ± SD. *p* < 0.05 were considered statistically different. * *p* < 0.05, ** *p* < 0.01, *** *p* < 0.001.

To further analyze the visual outcomes, patients were divided into four groups based on the number of DEX implants (one, two, three, and more than three). Results showed that VA did not change significantly after multiple DEX implants, but a gradual regression to baseline values was noted (Figure 2a). However, CRT showed a significant decrease one month after each DEX implant (*p* < 0.001 vs. baseline at each time point; Figure 2b) in patients who received more than one implant. Notably, in eyes receiving only one DEX implant, the reduction in CRT from baseline was significant and this effect persisted for 3 and 6 months. 

### 3.4. Intraocular Pressure Increases as a Marker of Adverse Effects

**Figure 3:**
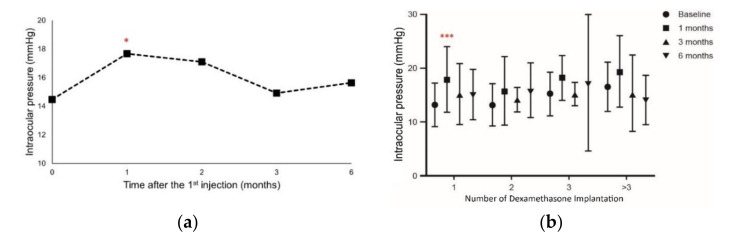
The change in intraocular pressure from baseline (**a**) after the 1st intravitreal dexamethasone implantation and (**b**) after the intravitreal dexamethasone implantation based on the number of implantations. Values represent mean ± SD. *p* < 0.05 were considered statistically different. * *p* < 0.05, *** *p* < 0.001.

To evaluate the occurrence of adverse effects due to DEX implants, IOP was assessed after each administration. We only observed an increase in IOP compared with baseline values at the first month after a single DEX implant (*p* < 0.05; Figure 3a). All eyes with elevated IOP were controlled with topical IOP-lowering eye drops for at least one month, without the need for IOP-lowering laser or incisional surgery. However, in the eyes that received more than one DEX implant, IOP did not change significantly at any time point throughout the whole study (Figure 3b). These results suggest that IOP increase due to a single DEX implant was only evidenced after one month, but this effect did not persist during the whole study. Thus, this indicates that DEX implants do not produce long-lasting significant adverse effects. If the patient had elevated IOP after receiving the DEX implant, they would not receive further implants as their IOP would require an evaluation period longer than one month. However, topical medication could be used to manage it.

## 4. Discussion

This study demonstrated that DEX implants improved VA and CRT, suggesting beneficial effects in the treatment of refractory ME in eyes with noninfectious uveitis. To date, there are few studies on DEX treatment in refractory uveitic ME. To the best of our knowledge, this is the first real-world study in Taiwan, where the etiology of uveitis is different from that in Western countries, with a higher incidence of juvenile idiopathic arthritis [16], Vogt–Koyanagi–Harada syndrome, and Behcet’s disease [18]. Although some of our findings have been reported previously, the effect of dexamethasone in refractory uveitic ME has not been well investigated in Asian populations, which may have a different response to steroid treatment. In addition, this was a real-world study with a long follow-up period and included patients with multiple DEX implants. 

In 2011, the Ozurdex HURON study group reported that VA improved after a single DEX implant in eyes with noninfectious uveitis, and that improvement was sustained for up to six months [19]. Later, investigators found that intravitreal DEX implants were effective in treating refractory uveitic ME [20], and in a multicenter, retrospective cohort study using DEX implants in eyes with noninfectious uveitis, a significant improvement in VA, central foveal thickness, and vitreous haze was reported, and the effects lasted for at least six months [21]. Other studies described positive outcomes one month after DEX implants, which remained for six months [10,19,22]. However, a shorter recurrence interval (five months) was reported in vitrectomized eyes with uveitic ME, and a DEX re-implantation interval between four and six months was suggested in this condition [23,24]. In accordance, our results showed that VA significantly increased one and two months after a single DEX implant administration and that this effect remained for six months. However, when patients were divided into different subgroups based on the number of implants, VA did not show significant differences between groups with more than one implant. This might be due to the lower number of eyes in each subgroup and a broader variation in VA, though an improving trend and the gradual regression of VA to baseline values was evidenced after DEX implants. 

Changes in VA are mainly related to changes in CRT [25,26]. Numerous clinical studies have demonstrated that CRT improves after DEX implant [27,28,29]. In eyes with uveitic ME, mean CRT was reduced after DEX implants with respect to baseline values, and that improvement was sustained for up to six months [14,30]. In this regard, our results on CRT are in agreement with previous studies that demonstrated that DEX implants had a significant effect on reducing CRT in eyes with uveitis [10,25,31]. The finding of a decreased CRT correlates with the improvement in VA observed in the eyes of the patients one month after the DEX implant. 

Although the observed effect was different one and two months after the DEX implant, we observed a trend in VA improvement that continued for up to six months. These results may be due to the small case number in late follow-up visits. On the other hand, CRT remained significantly low six months after DEX implants. For patients with a single injection, we have followed the biomarkers for up to 24 months (shown in Supplementary Figure S1), and found the CRT improvement could last for 24 months. However, the trend in VA improvement was not observed after the 6th month. Because patients with cataracts were not excluded from the study, the progression of cataracts may have affected VA results in late follow-up visits. Additionally, we did not compare the changes in cataract condition due to the retrospective nature of the study. In this regard, patients may be followed by different ophthalmologists in a single center, and it is known that the cataract grading depends on the ophthalmologist’s criteria.

In patients with a single implant, remission of macular edema was observed in 20 of 24 eyes (83.3%) during the follow-up period. In patients with multiple implants, remission of macular edema was observed in 20 of 28 eyes (71.4%). For eyes with recurrent macular edema, patients were either lost to follow-up, refused multiple implants, or, because of financial difficulties, dexamethasone implants were not continued. All the eyes in this study with multiple implants had therapeutic responses during the previous implant treatment based on our multiple-implantation criteria. For patients observed to have a good response to the previous dexamethasone implantation, we do suggest consideration of a repeat injection. However, the maximal number of implants has not been investigated. As for determination of responses, the investigation to predict biomarkers for the recurrence of uveitic macular edema is ongoing

DEX is a well-known corticosteroid widely used in the treatment of inflammatory diseases, including uveitis. Systemic and topical administration of corticosteroids and nonsteroidal anti-inflammatory drugs are common treatments and have proved effective in uveitic ME [32]. However, systemic steroids are frequently associated with multiple adverse effects [24]. Adverse side effects due to systemic steroid therapy include hyperglycemia, arterial hypertension, and weight gain, and they can increase IOP and cause cataracts [24]. However, in a three-year clinical trial, DEX implants showed a transient and controlled but not cumulative increase in IOP in eyes from patients with diabetic ME [33]. In accordance, DEX implants produced a rise in IOP within the first two weeks [34] and during the two months post-injection [35], but gradually decreased after 12 months. In line with these findings, our results demonstrated that IOP increases were observed only at 1 month after DEX administration, and the IOP could be controlled afterward. In our study, a non-invasive medical intervention was performed to control IOP and to treat elevated IOP. Given that transient IOP elevation may still be harmful in some patients, especially to treat those with pre-existing glaucoma, a close and regular IOP follow-up remains crucial after DEX implants, mainly two months after the injection.

To avoid inter-eye correlations, we performed a subgroup analysis which included only one eye from each patient (shown in Supplementary Figure S3). The results from the subgroup had comparable findings with the overall group using the eye as the unit. It may indicate that the effect of inter-eye correlations could be alleviated in the study analyzed using the longitudinal model using the eye as the unit of analysis [36].

DEX demonstrated efficacy in noninfectious uveitis in the HURON clinical trial, as evidenced by controlled intraocular inflammation for at least six months, together with a significant improvement in VA and decreased vitreous haze score and central foveal thickness [16]. Although the observed effects of multiple implant administrations were similar to those obtained solely with the first implant [36], the recommended interval between administrations is four to six months [24]. This may be reasonable in terms of efficacy, therapy duration, and the risk of adverse effects. Close follow-up and monitoring of adverse effects such as elevation of IOP and formation of cataracts were also emphasized in patients with multiple DEX implants [37].

However, there are limitations to this study inherent to its retrospective nature. In addition, some patients did not come back for follow-up, and the study had a small number of cases, especially after subdivision into smaller groups. The small number may affect the statistical significance, though the effect of repeated DEX implants appeared to be similar to the first injection. In our study, we did not include any power analysis due to our study design and population entity. In this regard, the population may be limited in case numbers compared to other ocular conditions; however, we included all eyes receiving DEX implants for refractory uveitic macular edema. Thus, the total number of eyes in the present study was higher when compared to similar previous studies [14,15]. Therefore, the results may still be able to demonstrate the real-world data for patients with uveitic macular edema treated with DEX implants. For IOP, results might exhibit a selection bias because the patients with elevated IOP after injection would not further receive DEX implants. The comparison of systemic side effects, as well as the analyses of systemic anti-inflammatory treatment, were not performed, resulting in the absence of potential/probable correlations in eyes that did not respond to DEX implants. Still, our study provides real-world results of intravitreal DEX implants in eyes with refractory ME in noninfectious uveitis. In addition, most studies on uveitic ME treated with DEX implants are short-term, retrospective studies with a limited number of eyes. Randomized studies are needed to evaluate the use of multiple DEX implants and to assess the long-term effects of repeated implants on IOP and the consequent cataract formation. Additionally, it is necessary to determine whether early treatment with DEX implants may be beneficial in reducing the adverse effects associated with systemic immunosuppressive treatments.

## 5. Conclusions

In conclusion, DEX implants are effective for eyes with refractory uveitic ME. However, while visual acuity appears to remain stable over the six months following an injection, the improvement in CRT seen in the first month fades over time.

## Figures and Tables

**Table 1:** Baseline demographics of patients treated with intravitreal dexamethasone implantation.

Characteristics	Value
No. of patients	37
No. of eyes	52
Age (years), mean ± SD (medium, min–max)	39.3 ± 18.4 (39, 11–74)
Follow-up time (months), mean ± SD (medium, min–max)	11.5 ± 6.9 (11, 3–24)
Sex, no. of patients (%)	
Female	27 (73.0)
Male	10 (27.0)
Disease, no. of eyes (%)	
Idiopathic uveitis	18 (34.6)
Behcet’s disease	9 (17.3)
Juvenile idiopathic arthritis	7 (13.5)
Vogt–Koyanagi–Harada syndrome	5 (9.6)
Acute anterior uveitis	5 (9.6)
Sarcoidosis	3 (5.7)
Sympathetic ophthalmia	2 (3.8)
Ulcerative colitis	2 (3.8)
Multiple sclerosis	1 (1.9)
Treatment prior to dexamethasone implantation, no. of eyes (%)	
Systemic steroid therapy	49 (94.2)
Topical steroid eye drops	19 (36.5)
Posterior sub-tenon triamcinolone injection	41 (78.8)
Disease-modifying antirheumatic drugs	34 (65.4)
Trabeculectomy, no. of eyes (%)	1 (1.9)
Pseudophakia, no. of eyes (%)	33 (64.7)
No of dexamethasone implantation, no. of eyes (%)	
1	24 (46.2)
2	15 (28.8)
3	7 (13.5)
>3	6 (11.5)
Baseline LogMAR VA, mean ± SD (medium, min–max)	0.81 ± 0.35 (0.82, 0.30–2.00)
Baseline IOP (mmHg), mean ± SD (medium, min–max)	13.3 ± 4.1 (13, 6–25)
Baseline CRT (µm), mean ± SD (medium, min–max)	507.5 ± 121.7 (512, 267–792)

SD: standard deviation, VA: visual acuity, IOP: intraocular pressure, CRT: central retinal thickness.

## Data Availability

The data presented in this study are available on request. The data are not publicly available due to the data security policy of Chang Gung Memorial Hospital.

## Supplementary Materials

The following are available online at https://www.mdpi.com/article/10.3390/jcm10173762/s1, Figure S1: The 24-month longitudinal change of (a) LogMAR visual acuity, (b) central retinal thickness, and (c) intraocular pressure in patients received single dexamethasone injection. * *p* <0.05, ** *p* <0.01, *** *p* <0.001; Figure S2: The Q-Q plots and results of Shapiro–Wilk test in patient’s baseline LogMAR visual acuity, central retinal thickness, and intraocular pressure.; Figure S3: The change in (a) LogMAR visual acuity, (b) central retinal thickness, and (c) intraocular pressure in subgroup with 37 eyes from the 37 patients. * *p* <0.05, *** *p* <0.001.

## Abbreviations

CRT	central retinal thickness
DEX	dexamethasone
DMARD	disease-modifying antirheumatic drug
IOP	intraocular pressure
ME	macular edema
OCT	optical coherence tomography
VA	visual acuity
