# Supplementary material for: Intravitreal Dexamethasone Implants for Refractory Macular Edema in Eyes with Noninfectious Uveitis
Source: J Clin Med. 2021 Aug 24;10(17):3762. doi: 10.3390/jcm10173762 (PMC8432099; doi:10.3390/jcm10173762)
Supplement: Supplementary file 1 [file jcm-10-03762-s001.zip › jcm-1290257-supplementary.pdf]

Supplementary Materials

The following are available online at www.mdpi.com/xxx/s1, Figure S1: The 24-month longitudinal change of (a) logMAR visual acuity, (b) central retinal thickness, and (c) intraocular pressure in patients who received single dexamethasone injection. *p <0.05, **p <0.01, ***p <0.001; Figure S2: The Q-Q plots and results of Shapiro-Wilt test in patient's baseline logMAR visual acuity, central retinal thickness, and intraocular pressure.; Figure S3: The change in (a) LogMAR visual acuity, (b) central retinal thickness, and (c) intraocular pressure in subgroup with 37 eyes from the 37 patients. *p <0.05, ***p <0.001.

Figure S1

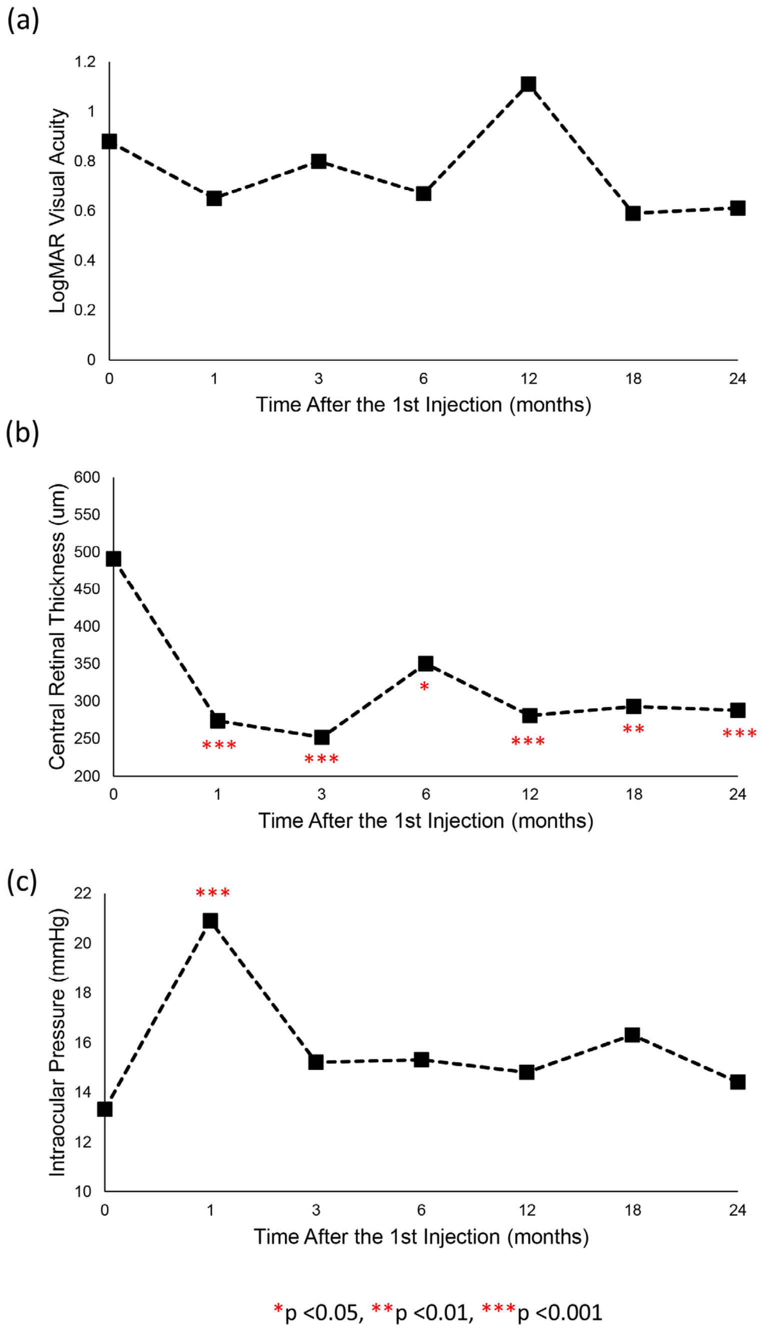

Figure S1. The 24-month longitudinal change of (a) LogMAR visual acuity, (b) central retinal thickness, and (c) intraocular pressure in patients received single dexamethasone injection. * p <0.05, ** p <0.01, *** p <0.001.

Figure S2

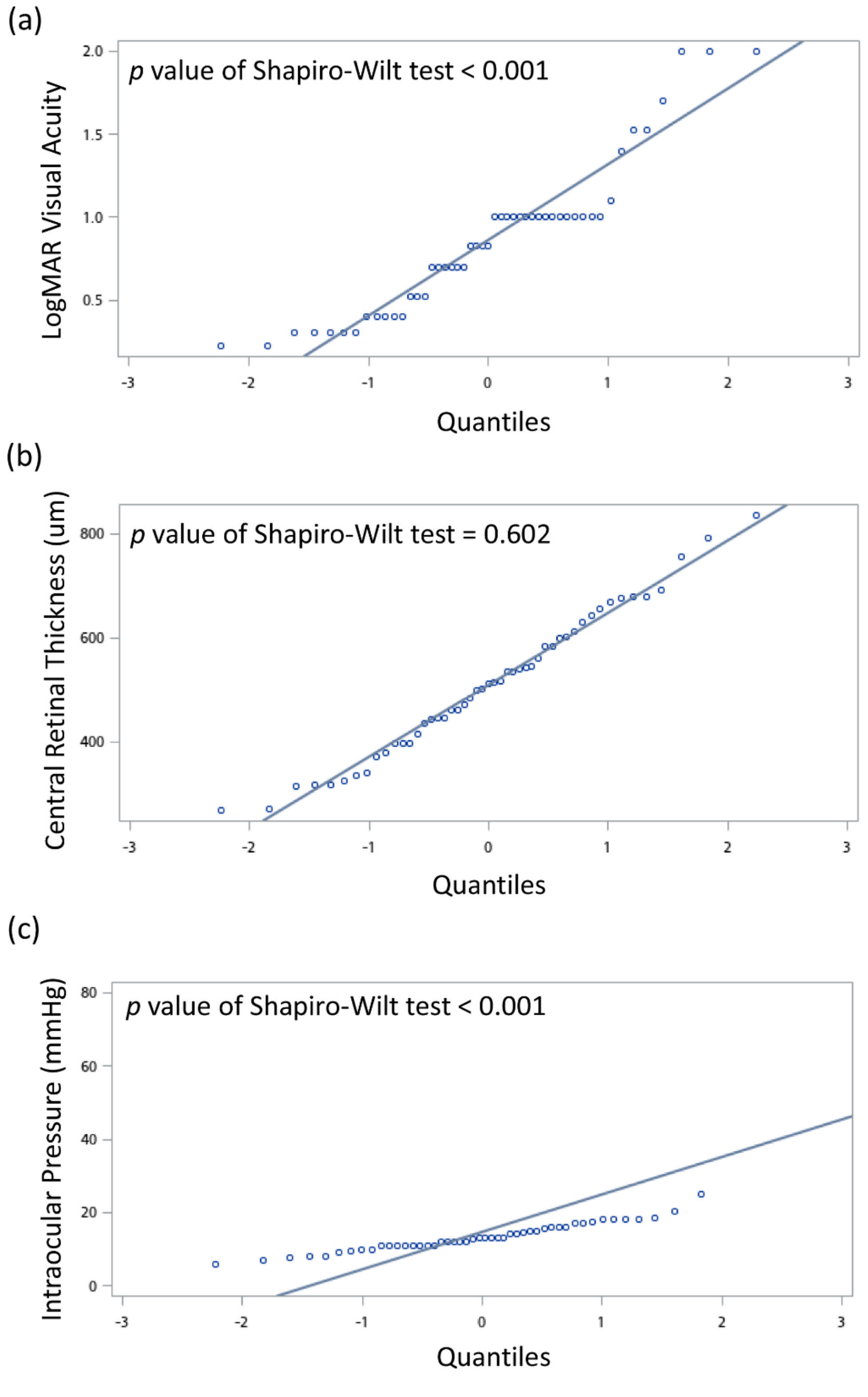

Figure S2. The Q-Q plots and results of Shapiro–Wilk test in patient's baseline LogMAR visual acuity, central retinal thickness, and intraocular pressure.

Figure S3

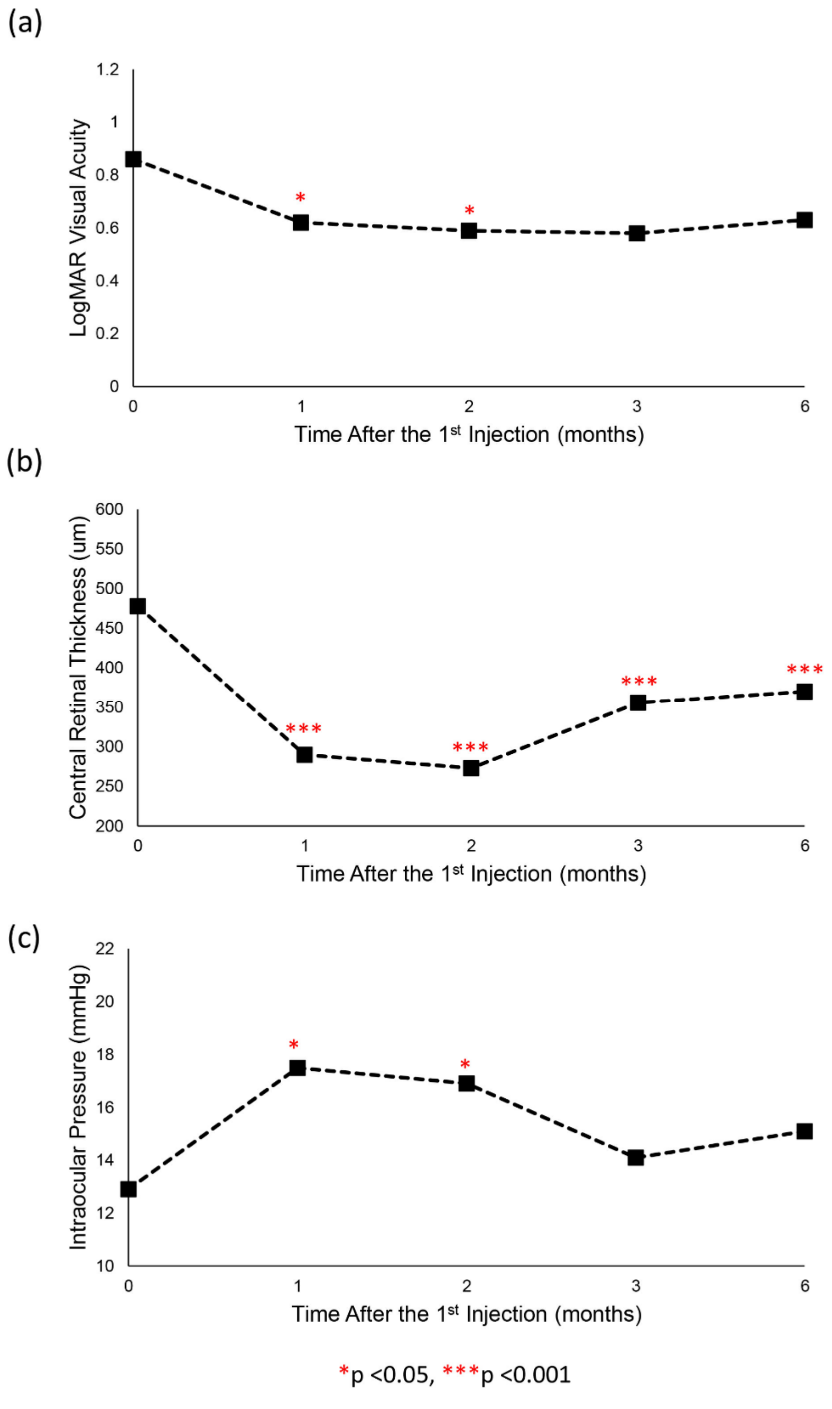

Figure S3. The change in (a) LogMAR visual acuity, (b) central retinal thickness, and (c) intraocular pressure in subgroup with 37 eyes from the 37 patients. * $p < 0.05$, *** $p < 0.001$.
